# Percolation Forces in Lung Inflammation: Determining the Path to Emphysema or Fibrosis

**DOI:** 10.3390/biomedicines14020281

**Published:** 2026-01-27

**Authors:** Jerome Cantor

**Affiliations:** School of Pharmacy and Allied Health Sciences, St John’s University, Queens, NY 11439, USA; cantorj@stjohns.edu

**Keywords:** pulmonary emphysema, pulmonary fibrosis, crosslinking, percolation networks, emergent phenomena

## Abstract

The dichotomous outcomes of chronic lung inflammation, represented by either pulmonary emphysema or interstitial fibrosis, involve poorly understood overlapping mechanisms. Recent insights from network theory suggest that percolation phenomena, coupled with the dynamics of extracellular matrix crosslinking, play an important role in determining these divergent pathological trajectories. This review examines how critical percolation thresholds at which local damage or repair transitions to system-wide structural failure or rigidification determine the changes in lung tissue during chronic inflammation. We examine the mechanisms of collagen and elastin crosslinking, the feedback loops that amplify initial perturbations, and the threshold behaviors that push inflamed lung tissue toward either emphysematous destruction or fibrotic consolidation. Understanding these percolation-dependent transitions provides new insights into why similar inflammatory insults can produce opposite structural outcomes and suggests novel therapeutic strategies targeting the crosslinking mechanisms that underlie these critical transitions.

## 1. Introduction

**Figure 1:**
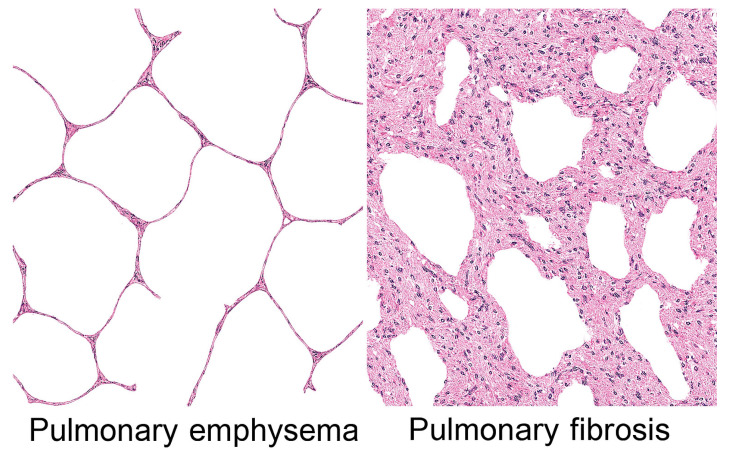
Pulmonary emphysema is characterized by distended and ruptured alveolar walls, whereas interstitial fibrosis is marked by cellular proliferation and fibrosis, resulting in pronounced thickening of alveolar walls.

The human lung is a complex biological network involving a delicate balance between extracellular matrix (ECM) proteins, cellular populations, and mechanical forces that maintains both structural integrity and functional compliance. When this equilibrium is disrupted by chronic inflammation, the lung can follow one of two dramatically different pathological paths: emphysema, characterized by progressive destruction of alveolar walls and airspace enlargement, or interstitial fibrosis, marked by excessive collagen deposition and tissue stiffening (Figure 1).

The apparent paradox involving similar inflammatory stimuli, such as cigarette smoke, environmental pollutants, or autoimmune processes that result in either tissue destruction or excessive tissue formation, has remained poorly understood [1]. Traditional explanations have focused on differences in inflammatory cell populations, cytokine profiles, or genetic susceptibility [2,3]. However, these factors alone cannot fully explain why inflammation sometimes leads to a self-propagating wave of tissue destruction, while at other times it culminates in progressive fibrotic consolidation. Continuum models, which assume uniform tissue properties, cannot fully account for the nonlinear, abrupt, and regionally clustered progression observed in either disease. These limitations have motivated the application of network-based frameworks capable of capturing how local structural changes propagate to whole-organ behavior.

Percolation theory, originally developed in statistical physics to describe connectivity transitions in disordered networks, offers a rigorous mathematical foundation for understanding the multiscale behavior of the lung parenchyma [4]. The alveolar interstitium is a highly interconnected, load-bearing network of elastin, collagen, and proteoglycans. Its mechanical integrity depends not only on the material properties of individual fibers but also on the topology of their connections. This architecture makes the lung uniquely susceptible to percolation-like transitions, in which small, spatially localized changes in connectivity can trigger large-scale alterations in mechanical function. Below a critical point, damage remains localized and contained, but above this threshold, damage or connectivity can propagate throughout the entire system. It is proposed that percolation phenomena govern whether inflammatory damage cascades into either emphysema or interstitial fibrosis.

Percolation theory also provides a mechanistic bridge between structural remodeling and cellular behavior. As the parenchymal network approaches its critical threshold, stress distributions become increasingly heterogeneous. Local stress concentrations accelerate elastin fracture in emphysema and promote fibroblast activation in fibrosis, creating positive feedback loops that drive disease progression [5]. This mechanochemical coupling suggests that percolation is not merely a descriptive framework but a mechanistic one, linking microstructural connectivity to biochemical signaling and tissue remodeling.

Central to this process is extracellular matrix crosslinking, which alters tissue mechanical properties and resistance to degradation [6]. This concept is supported by a study using beta-aminopropionitrile, an elastin and collagen crosslink inhibitor, to modify cadmium chloride-induced lung injury [7]. Treatment with this agent produced pulmonary emphysema instead of interstitial fibrosis, emphasizing the multiscale evolution of lung disease, involving molecular changes that are reflected by remodeling of lung structure.

The current review further explores this phenomenon by integrating crosslink formation with percolation forces, highlighting the development of mechanical and biochemical feedback loops that can steer tissue toward either degenerative or fibrotic outcomes. Based on this analysis, we hypothesize that pulmonary emphysema and interstitial fibrosis may be two sides of the same coin, involving probabilistic outcomes related to percolation thresholds.

## 2. Percolation Theory and Biological Networks

Percolation theory provides a rigorous, mechanistically grounded framework for understanding the pathogenesis of pulmonary emphysema and interstitial fibrosis. By treating the lung as a complex, interconnected network rather than a homogeneous continuum, it explains how spatially localized microstructural changes can produce nonlinear, emergent alterations in whole-organ mechanics. This approach unifies the pathophysiology of two seemingly opposite diseases, clarifies the behavior of mixed phenotypes such as CPFE, and offers a foundation for integrating imaging, biomarkers, and mechanobiology into a coherent multiscale model of lung disease.

The theory describes how local connections in a network determine global connectivity and system-wide properties [8]. It provides a framework for analyzing how randomly occupied sites or bonds in a lattice affect the emergence of interconnected spanning clusters that extend across the entire system. Below a critical occupation probability (the percolation threshold), only small, isolated clusters exist. Above this threshold, spanning clusters suddenly emerge that fundamentally change the network’s properties.

In biological tissues, percolation concepts apply to multiple scales and contexts. At the molecular level, crosslinks between matrix proteins form a network whose connectivity determines mechanical properties [9]. At the cellular level, the presence or absence of cells maintaining tissue architecture affects structural integrity [10]. At the tissue level, the continuity of alveolar walls determines whether the gas exchange surface area is preserved or lost.

The lung extracellular matrix forms a three-dimensional percolation network where collagen fibers, elastin filaments, proteoglycans, and other matrix components create a mechanically integrated structure [11]. In healthy lung tissue, this network operates above the percolation threshold where the matrix is fully connected, creating a continuous mechanical scaffold that distributes stress uniformly and resists local damage. Crosslinks between matrix proteins are crucial for maintaining this connected state, providing the covalent bonds that link individual proteins into a functional network [12].

**Figure 2:**
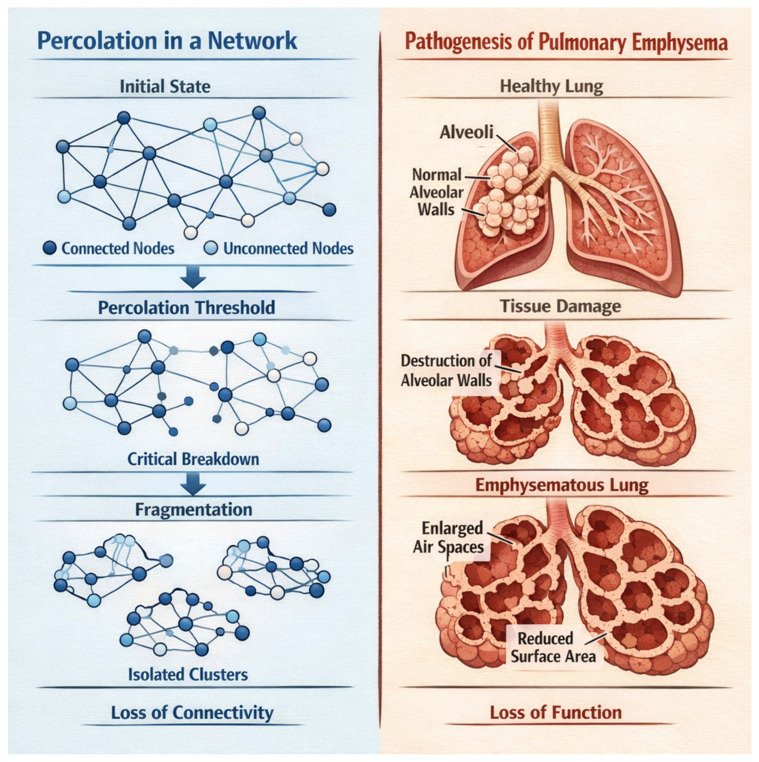
The fragmentation of percolation clusters below a critical threshold corresponds to the rupture of alveolar walls in pulmonary emphysema. This loss of connectivity impairs the distribution of mechanical forces, resulting in air trapping and reduced gas exchange.

When inflammation introduces proteolytic enzymes, oxidative stress, and mechanical forces that damage matrix components, the tissue approaches a critical percolation threshold from above. If crosslink density falls below a critical value, or if too many structural connections are severed, the network fragments [13]. This fragmentation represents a percolation transition involving a loss of global connectivity and the emergence of mechanical instability. In emphysema, this downward percolation transition leads to progressive tissue destruction (Figure 2).

**Figure 3:**
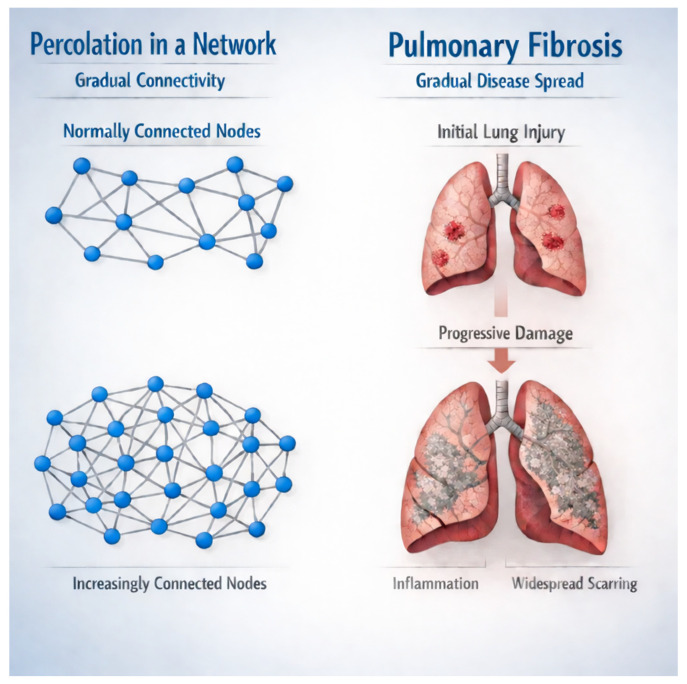
The increased crosslinking in pulmonary fibrosis corresponds to enhanced percolation connectivity, which increases mechanotransduction-induced fibrogenesis.

Conversely, excessive crosslinking during repair processes can drive tissue toward a different outcome. When crosslink density significantly increases above normal levels, the matrix becomes rigid and resistant to normal remodeling [14]. This upward transition through a rigidity percolation threshold creates a mechanically stiff network that resists degradation and continues to accumulate additional matrix. In fibrosis, this transition manifests as progressive tissue stiffening and loss of functional compliance (Figure 3).

The concept of percolation thresholds helps explain why the progression of lung disease often appears non-linear. Patients may maintain relatively stable lung function despite ongoing inflammation until a critical threshold is crossed, after which rapid deterioration occurs. This threshold behavior reflects the fundamental physics of percolation transitions in which small changes near critical points can have disproportionate effects on system-wide properties.

With regard to pulmonary emphysema, the primary percolation process is a loss of connectivity, where the loss of septal attachments causes cluster fragmentation. Once the system crosses the connectivity threshold, large airspaces emerge, and the network becomes flaccid, unable to transmit stress uniformly. Local stresses concentrate at remaining walls, causing further alveolar wall rupture and loss of connectivity.

In the case of pulmonary fibrosis, excess collagen deposition adds bonds and increases the stiffness of existing bonds. The activity of crosslinking enzymes increases constraint density. Once the system crosses a critical threshold, the percolation network becomes over-constrained, resisting deformation. Stress no longer distributes smoothly; instead, it funnels into rigid tracts, inducing further fibroblast activation.

The relationship between crosslinking connectivity and disease state may be expressed as follows:

Let C(t) represent the time-dependent crosslinking connectivity of the ECM. The trajectory of lung injury can be modeled as follows:

Emphysema, if C(t) < Ccrit;

Fibrosis, if C(t) > Ccrit;

Mixed/Transitional, if C(t) ≈ Ccrit.

Here, Ccrit is the percolation threshold for mechanical coherence. This threshold is influenced by:

Crosslinking enzyme activity: lysyl oxidase (LOX).

Matrix composition: collagen vs. elastin ratios.

Mechanical loading: cyclic stretch, shear stress.

Inflammatory milieu: cytokines and oxidative stress.

Elastic fiber integrity: fracture susceptibility under hypercrosslinking.

Feedback and Bifurcation.

Once the system crosses Ccrit, feedback loops reinforce the trajectory:

Below the threshold: Protease activity dominates, further degrading matrix and suppressing crosslinking, thereby driving emphysema.

Above the threshold: Fibroblast proliferation and LOX expression enhances crosslinking and induces fibrosis.

**Figure 4:**
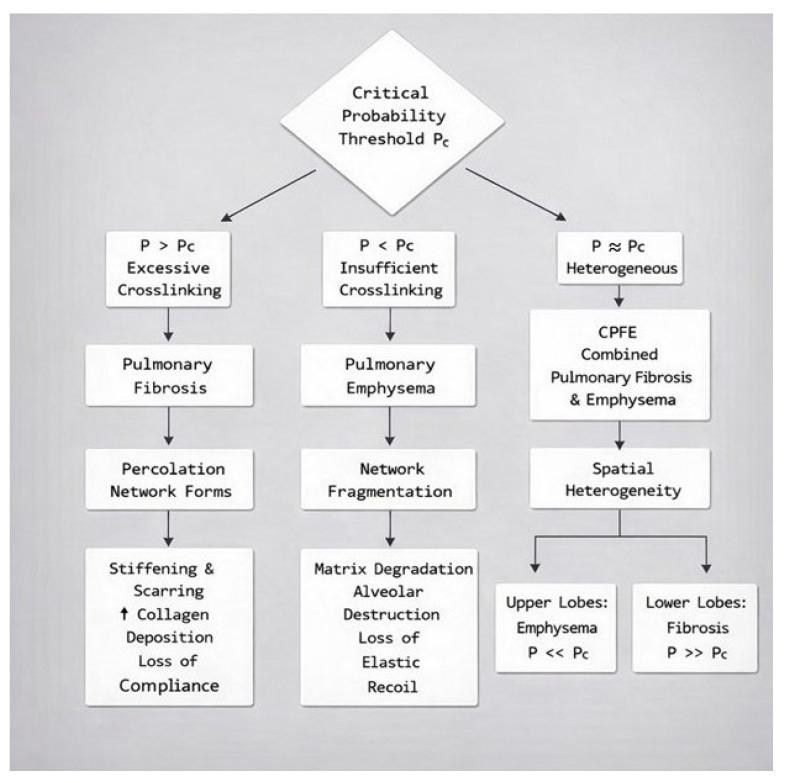
Flow chart representing the divergence of lung structural changes based on the relationship between crosslink density and the critical probability threshold associated with percolation network connectivity.

This connectivity-driven bifurcation offers a mechanistic explanation for how similar initial injuries (e.g., smoke exposure, viral insult) can diverge into distinct pathological outcomes based on emergent ECM topology (Figure 4).

## 3. Mathematical Relationship Between Crosslinking and Percolation Connectivity

The lung’s ECM can be modeled as a three-dimensional network where nodes represent crosslinking sites between matrix molecules, and bonds represent the structural proteins (collagen and elastin fibers). Bond occupation probability (p) represents the fraction of intact, functional connections. The elastic modulus (E) of the lung parenchyma depends critically on network connectivity and pulmonary emphysema develops when the ECM network loses mechanical continuity. Near the percolation threshold (p_c_) from below

E ~ (p_c_ − p)^t^

where t is the conductivity exponent. As the network approaches fragmentation, mechanical stiffness begins to collapse and a small additional loss of connectivity causes disproportionate mechanical failure. Compliance (C), which is inversely proportional to E, increases dramatically, as reflected by a greater change in volume (V) in response to inspiratory pressure (P).

C = dV/dP ∝ (p_c_ − p) ^− t^

The progression toward emphysema can be modeled as a time-dependent bond removal process. If proteases degrade bonds at rate kdeg and repair occurs at rate krep, the time evolution of connectivity is as follows:

dp/dt = krep(1 − p) − kdeg · p

**Figure 5:**
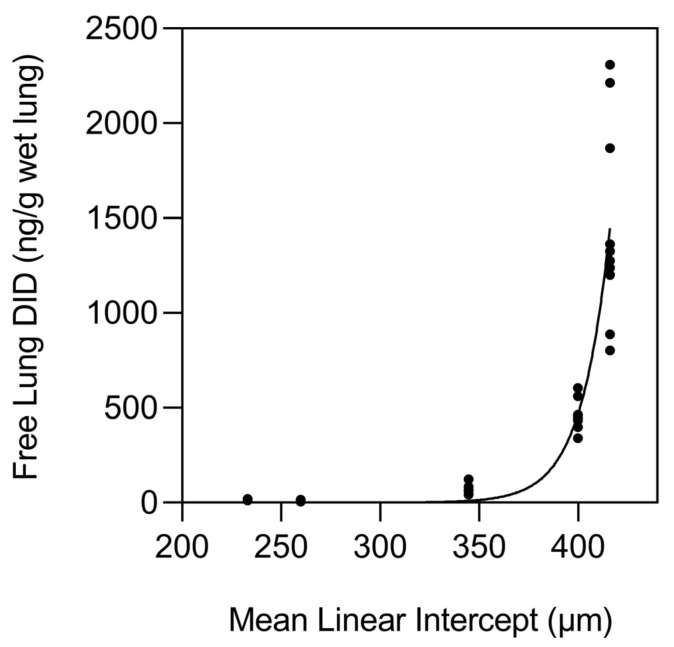
The release of free desmosine from elastic fibers is exponentially increased as alveolar diameter, as measured by the mean linear intercept method, exceeds 400 µm. This process is associated with the movement of percolation forces below the critical threshold for connectivity. Reprinted with permission [15].

This mathematical construct is supported by studies of normal and emphysematous human postmortem lungs which showed an exponential increase in elastin breakdown, as measured by the release of desmosine crosslinks, when alveolar diameter exceeded 400 µm (Figure 5) [15].

Pulmonary fibrosis represents the opposite extreme: pathological increases in network connectivity through excessive crosslinking. This process is primarily due to the activity of the lysyl oxidase (LOX) family of enzymes. They catalyze the oxidative deamination of lysine and hydroxylysine residues in collagen and elastin, forming reactive aldehydes (allysine) that spontaneously condense to form covalent crosslinks. The relationship between crosslink density (crosslinks per unit volume) and LOX activity may be expressed as follows:

$$\rho_{XL}=\rho_{XL(0)}+kA_{LOX}$$

where

$\rho_{XL}$ = crosslink density;

$\rho_{XL(0)}$ = baseline crosslink density;

$A_{LOX}$ = LOX activity;

k = proportionality constant reflecting collagen availability and catalytic efficiency.

**Figure 6:**
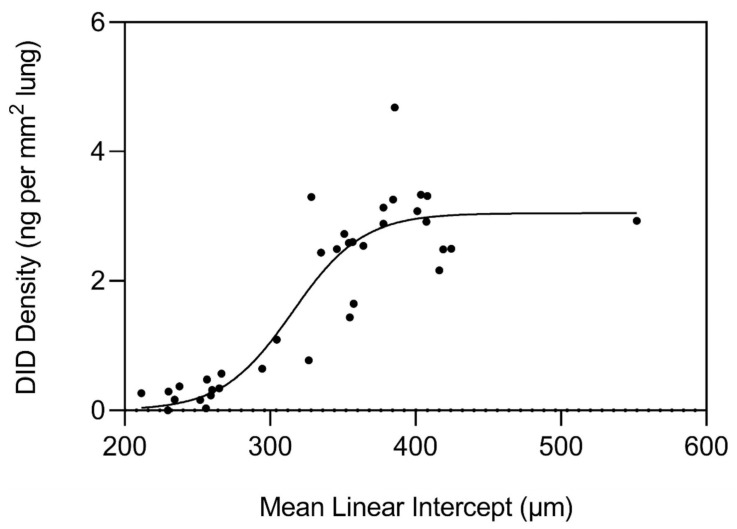
Crosslink density in human emphysema shows a sinusoidal change in crosslink density as alveolar diameter increases. However, as airspace size exceeds 400 µm, this process levels off, consistent with a divergence from the continued increase in crosslinking associated with pulmonary fibrosis. Reprinted with permission [15].

As the disease process differentiates toward interstitial fibrosis, TGF-β signaling upregulates LOX expression while simultaneously increasing collagen synthesis, resulting in a rapid increase in crosslink density [16]. During the initial stages of this process, greater crosslink density is also a feature of pulmonary emphysema, as reflected by measurements of normal and emphysematous human postmortem lungs (Figure 6) [15]. However, the repair process in emphysematous lungs eventually undergoes a decompensatory phase involving the release of desmosine as airspace enlargement progresses (Figure 5), whereas fibrotic lung disease involves continued deposition of extracellular matrix, resulting in an increase in crosslink density. Based on these findings, therapeutic regulation of LOX activity at an early stage of lung injury could restore normal percolation processes.

This divergence in disease outcomes most likely represents the emergence of multiscale processes that alter the balance of percolation forces. In pulmonary emphysema, an abrupt change in the competition between mechanical stress and crosslinking results in a loss of percolation connectivity. Conversely, a different temporal and spatial pattern of mechanical force disruption in pulmonary fibrosis may induce epithelial–mesenchymal transition that results in fibrogenesis and percolation rigidity.

While this percolation model provides a valuable tool for describing network failure or rigidification, it is based on certain assumptions that may not be applicable at all levels of scale. The representation of the lung as a homogeneous lattice may not adequately reflect the spatial heterogeneity of the pulmonary parenchyma. Alveolar units differ in size, shape, and mechanical coupling, and may be subject to hierarchical relationships that are not fully captured by the model [5]. The role of critical probability thresholds is most valid at lower levels of scale, where binary processes, such as the presence or absence of crosslinking, are primary determinants of disease progression [17]. At higher levels, the model may not adequately represent cellular processes and feedback loops that regulate disease progression and structural outcomes. Nevertheless, the emphasis on crosslinking as a fundamental mechanism in the development of either pulmonary emphysema or interstitial fibrosis provides a promising theoretical framework for designing more effective therapeutic interventions.

## 4. Extracellular Matrix Crosslinking: Molecular Mechanisms and Types

### 4.1. Enzymatic Crosslinking

The most important physiological crosslinking enzymes are those comprising the LOX family, which includes LOX and four LOX-like proteins (LOXL1-4). These copper-dependent amine oxidases catalyze the oxidative deamination of lysine and hydroxylysine residues in collagen and elastin [18]. This process generates reactive aldehyde groups, which spontaneously condense with other aldehydes or with unmodified lysine residues to form Schiff bases and aldol condensation products. These initial labile crosslinks subsequently undergo further reactions to form stable, mature crosslinks.

**Figure 7:**
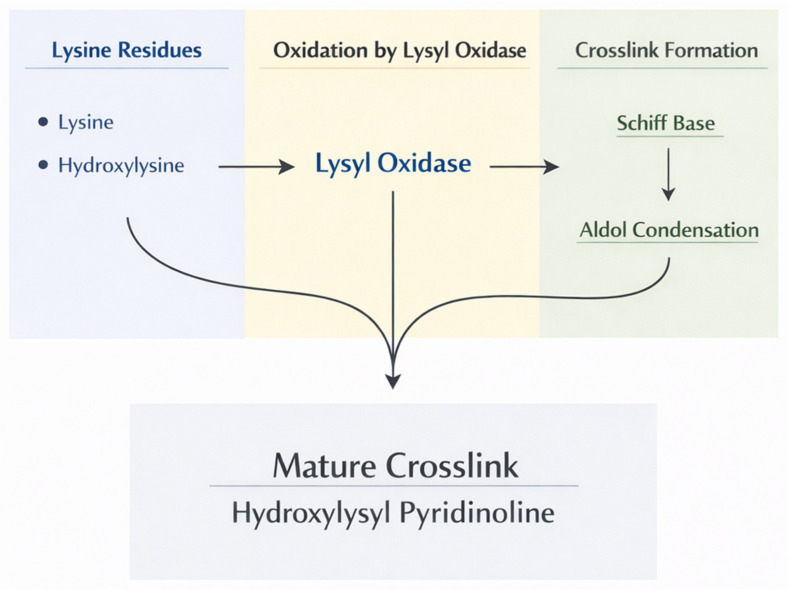
Flow chart showing the pathways associated with the formation of mature trivalent collagen crosslinks.

In collagen, LOX-mediated crosslinking produces several types of mature crosslinks. Divalent crosslinks such as dehydrohydroxylysinonorleucine (deH-HLNL) and hydroxylysinonorleucine (HLNL) form from the condensation of one allysine with one hydroxylysine or lysine. These can further mature into trivalent crosslinks, such as hydroxylysyl-pyridinoline (HP) and lysyl-pyridinoline (LP), which link three collagen chains. The nature and density of these crosslinks profoundly affect the mechanical properties and resistance to proteolytic degradation of collagen fibrils (Figure 7).

**Figure 8:**
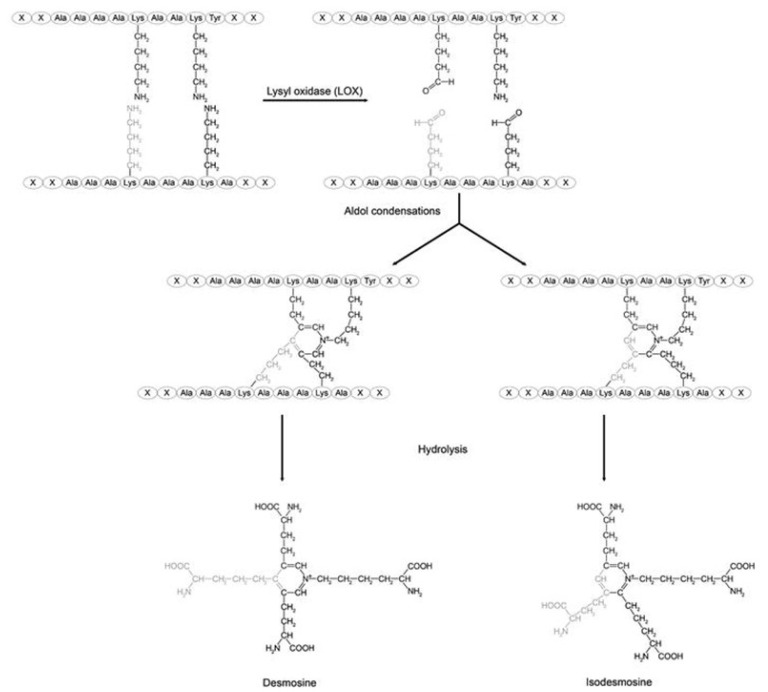
The formation of the unique desmosine and isodesmosine crosslinks are formed by the condensation of four lysine residues. Reprinted with permission of Creative Commons (https://creativecommons.org/licenses/by-sa/4.0/ (accessed on 10 February 2025)).

Elastin crosslinking by LOX produces the unique desmosine and isodesmosine crosslinks which link four polypeptide chains (Figure 5) [19]. These tetrafunctional crosslinks are essential for elastin’s rubber-like mechanical properties, allowing reversible deformation during breathing [20]. The density and pattern of elastin crosslinking directly determine tissue elastic recoil and compliance (Figure 8).

Tissue transglutaminases (TG) represent another family of crosslinking enzymes [21]. These calcium-dependent enzymes catalyze the formation of ε(γ-glutamyl)lysine isopeptide bonds between glutamine and lysine residues. Transglutaminase-2 (TG2), the most studied family member, crosslinks various matrix proteins, including fibronectin, collagen, and other ECM components. During inflammation, TG2 expression increases markedly, and aberrant transglutaminase activity contributes to excessive crosslinking in fibrotic tissue [22].

### 4.2. Non-Enzymatic Crosslinking

Non-enzymatic glycation produces advanced glycation end-products (AGEs), which form spontaneous crosslinks between proteins [23]. The initial Schiff base formed between polysaccharides and amino groups undergoes rearrangement, followed by complex oxidative and non-oxidative reactions, to produce stable AGE crosslinks. Common AGE crosslinks include N-ε-(carboxymethyl)lysine (CML), pentosidine, and glucosepane.

These crosslinks accumulate with age and are accelerated by diabetes, oxidative stress, and inflammation [24]. Unlike enzymatic crosslinks, AGEs form randomly rather than at specific sites, potentially creating crosslinking patterns that disrupt normal matrix organization [25]. AGE-modified proteins also bind to receptors (RAGE), triggering inflammatory signaling that can amplify tissue damage [26]. RAGE is highly expressed on type I alveolar epithelial cells, and its loss is associated with injury to these cells in pulmonary fibrosis, suggesting that diminished RAGE contributes to impaired epithelial repair and aberrant remodeling in this disease [26].

Additional crosslinking occurs when reactive oxygen and nitrogen species generate free radicals that interact with matrix proteins. Tyrosine residues can form dityrosine crosslinks through radical coupling reactions [27]. Lipid peroxidation products such as malondialdehyde and 4-hydroxynonenal can also form crosslinks between proteins. These oxidative crosslinks are particularly relevant in emphysema, where oxidative stress from cigarette smoke and inflammatory cells is pronounced [28].

### 4.3. Crosslink Dynamics and Tissue Mechanics

Higher crosslink density generally increases the elastic modulus and reduces compliance. However, the relationship is complex because the type of crosslink matters. LOX-mediated enzymatic crosslinks occur at specific sites that optimize mechanical function, while non-enzymatic crosslinks form randomly and may create mechanical stress concentrations [13].

As collagen crosslinking increases, it becomes more resistant to matrix metalloproteinases (MMPs) and other proteolytic enzymes. This process creates a positive feedback loop in fibrosis: excessive crosslinking makes the matrix resistant to regular turnover, leading to progressive matrix accumulation [11]. Conversely, in emphysema, the loss of normal crosslinks makes the remaining matrix more susceptible to proteolytic attack, accelerating tissue destruction [15].

The spatial distribution of crosslinks creates mechanical heterogeneity within tissues. Regions with high crosslink density become mechanical stress concentrators, while regions with low crosslink density become vulnerable failure points [8]. This heterogeneity is critical for percolation behavior, where the spatial variation in crosslink density determines whether the network has sufficient connectivity to maintain structural integrity or whether vulnerable regions can serve as focal points for catastrophic failure.

### 4.4. Genetic Factors in Crosslinking

Genetic factors associated with cellular dysfunction can influence crosslinking due to their multiscale effects on lung injury and repair. The MUC5B genetic variant drives excessive mucin expression and is associated with epithelial dysfunction and senescence, while short telomeres, common in telomere-related lung disease, further impair epithelial renewal and promote chronic injury responses [29,30]. Together, these vulnerabilities amplify profibrotic signaling from stressed bronchoalveolar epithelium, increasing secretion of TGF-β, LOX/LOXL enzymes, and reactive oxygen species. Telomere-mediated senescence also enhances the production of cytokines that recruit fibroblasts and skew them toward a highly crosslinked, myofibroblast phenotype [30]. In this genetically altered landscape, the ECM becomes progressively enriched in mature, stiff crosslinks, creating a mechanically rigid microenvironment that perpetuates fibroblast activation and locks the lung into a self-reinforcing cycle of fibrosis.

## 5. Emphysema: Degradative Percolation and Crosslink Loss

### 5.1. The Multiscale Effects of Decreased Crosslink Density

Desmosine crosslinks give elastic fibers their remarkable resilience, allowing tissues like lung parenchyma, skin, and blood vessels to stretch repeatedly without mechanical failure. These unique tetrafunctional crosslinks create a highly interconnected, three-dimensional network that resists both tensile overload and fatigue. Their multivalent architecture distributes strain across many elastin molecules simultaneously, preventing localized stress concentrations that would otherwise lead to microtears, fragmentation, or irreversible stiffening. When desmosine formation is impaired, or when oxidative or proteolytic processes degrade them, the elastic fiber network loses its recoil capacity, contributing to airspace distention and rupture.

This process involves a downward percolation transition involving progressive loss of structural connectivity. While inflammation and protease-antiprotease imbalance are recognized contributors, percolation concepts reveal why this damage propagates catastrophically once initiated. In particular, the loss crosslink density promotes subsequent multiscale changes in alveolar architecture, mechanical forces, and gas exchange.

### 5.2. Initiation: Local Crosslink Disruption

**Figure 9:**
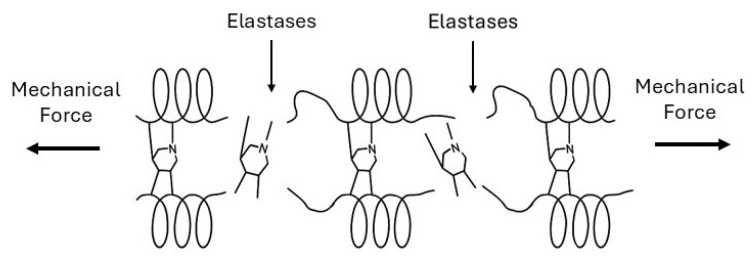
The loss of elastin crosslinks in pulmonary emphysema is the result of elastase activity combined with mechanical forces.

Cigarette smoke, the primary risk factor in the development in pulmonary emphysema, induces both direct oxidative stress and inflammatory cell recruitment, which disrupt extracellular matrix crosslinking [31]. Neutrophils and macrophages release elastases, matrix metalloproteinases, and reactive oxygen species that attack matrix proteins and their crosslinks. Oxidative modification of elastin crosslinks changes their structure and weakens the elastic fiber network [32]. When desmosine and isodesmosine undergo breakdown, the mechanical load redistributes to neighboring crosslinks, increasing their stress (Figure 9).

While collagen fibers are more resistant to proteolytic degradation than elastin, oxidative modification of lysine-derived crosslinks can also reduce their stability. The combination of proteolytic and oxidative damage progressively reduces crosslink density in localized regions.

### 5.3. Percolation Transition: Loss of Network Connectivity

As the fraction of intact bonds falls below a critical value, no connected pathways span the system, and mechanical load cannot be distributed uniformly throughout the system. In emphysematous lung tissue, this transition manifests as mechanical failure of alveolar walls. Individual alveolar walls, weakened by the loss of crosslinks and matrix degradation, rupture under normal breathing stresses. This process creates larger airspaces and redistributes mechanical stress to adjacent walls. The concentration of stress on remaining intact walls increases their susceptibility to failure, creating a positive feedback loop.

Computational models of alveolar mechanics demonstrate this behavior [32,33]. When the decrease in crosslink density reaches a certain threshold (based on network geometry), stress concentrations become so severe that breathing mechanics alone can propagate the rupture. The heterogeneous nature of crosslink loss is a critical feature of this process [8]. If crosslinks were uniformly reduced throughout the lung, the percolation threshold would require more extensive damage. However, inflammatory damage is spatially heterogeneous, with regions exposed to higher smoke concentration, greater inflammatory cell infiltration, or pre-existing structural weakness experiencing more severe crosslink loss. These vulnerable regions act as nucleation sites where percolation transitions first occur.

### 5.4. Propagation: Mechanical Amplification

Once local regions have crossed the percolation threshold and mechanical connectivity is lost, mechanical forces amplify the damage. Breathing mechanics create cyclic stresses that preferentially concentrate at the edges of damaged regions [34]. The boundary between intact tissue and emphysematous regions experiences stress concentrations that can be significantly higher than the baseline [35].

These stress concentrations accelerate crosslink breakage and matrix protein fatigue at the damage boundary. Collagen fibers experience increased mechanical load, and fatigue failure of crosslinks occurs even without additional proteolytic activity. This mechanical propagation explains why emphysema progresses even after smoking cessation [10].

The loss of elastic recoil in emphysematous regions also affects the global mechanics of the lungs. Normal expiration depends on elastic energy stored during inspiration. As emphysema destroys elastic tissue, the driving force for expiration diminishes, leading to air trapping and hyperinflation. Hyperinflation further increases stress on the remaining alveolar walls, creating a positive feedback loop that drives disease progression.

### 5.5. Crosslink Repair Failure

In healthy tissue, matrix damage triggers repair responses, including the synthesis of new matrix and crosslink formation. However, in emphysema, these repair mechanisms are insufficient. Several factors contribute to this condition, including oxidative stress that impair LOX activity and the presence of inflammatory mediators that disrupt the balance between matrix synthesis and degradation. Additionally, the mechanical environment in emphysematous regions may not provide appropriate signals for organized matrix deposition [36].

The failure to restore crosslink density above the percolation threshold allows the degradative process to continue. Unlike acute injuries that heal, emphysema represents a state in which the tissue remains below the critical connectivity threshold, unable to restore its mechanical integrity [37]. This irreversibility is a hallmark of percolation transitions: once the system has fragmented, restoring connectivity requires extensive reorganization that exceeds the tissue’s regenerative capacity.

## 6. Fibrosis: Rigidity Percolation and Excessive Crosslinking

### 6.1. Initiation: Aberrant Repair and Crosslinking

Pulmonary fibrosis typically begins with repetitive alveolar epithelial injury and abnormal wound healing [38]. Factors responsible for this process include occupational exposures, drugs, radiation, and autoimmune conditions. Following injury, type II alveolar epithelial cells undergo apoptosis or senescence, and myofibroblast proliferation and activation occur in the underlying interstitium [39].

Myofibroblasts are the primary cells involved in lung fibrogenesis, characterized by expression of α-smooth muscle actin and production of excessive extracellular matrix [40]. These cells synthesize collagen types I and III at rates significantly exceeding those of normal fibroblasts. Activated myofibroblasts also dramatically upregulate LOX and LOXL2 expression, increasing enzymatic crosslinking of newly deposited collagen [41].

Transforming growth factor-β (TGF-β), the primary regulator of fibrosis, drives this process. TGF-β induces myofibroblast differentiation, stimulates collagen synthesis, and upregulates LOX expression [42]. It also inhibits matrix degradation by reducing MMP expression and increasing tissue inhibitors of metalloproteinases [43]. These mechanisms shift the balance in favor of matrix accumulation and crosslinking.

### 6.2. Percolation Transition: Rigidity Threshold

As the crosslink density increases, the increasing rigid percolation network significantly alters the mechanical properties of the lung [13]. Computational models and experimental measurements show that lung tissue stiffness rapidly increases when crosslink density exceeds a critical threshold [44,45]. This process creates mechanical stress on cells, particularly alveolar epithelial cells, which can promote further epithelial injury and apoptosis. Matrix rigidity also activates mechanotransduction pathways in fibroblasts and myofibroblasts, promoting continued collagen production and crosslinking [46,47].

The mechanotransduction pathway linking matrix stiffness to continued fibrosis creates a positive feedback loop. Various transcription factors, which accumulate in the nucleus in response to stiff substrate, drive expression of pro-fibrotic genes responsible for synthesis of TGF-β, LOX, and collagen [42,48,49]. Thus, crosslinking-induced stiffness induces a self-propagating process that further drives the tissue further beyond the rigidity percolation threshold.

### 6.3. Crosslink Types in Fibrosis

The types of crosslinks in fibrotic tissue differ from those in normal lung. LOXL2, which is dramatically upregulated in fibrosis, generates crosslinks that may differ in structure and mechanical properties from LOX-mediated crosslinks [8,42]. Studies suggest that LOXL2 preferentially crosslinks newly synthesized collagen before proper fibril formation, potentially creating mechanically abnormal networks [50].

Transglutaminase-mediated crosslinks also accumulate in fibrotic tissue. TG2 expression increases in response to TGF-β, and TG2 crosslinks various matrix proteins into abnormally stable complexes [25]. These crosslinks are highly resistant to proteolytic degradation, contributing to the irreversibility of established fibrosis.

AGE crosslinks accumulate in fibrotic tissue, accelerated by inflammation and oxidative stress. These non-enzymatic crosslinks create additional mechanical rigidity and further resistance to degradation [25]. The random placement of AGE crosslinks may also disrupt normal matrix architecture, creating regions of extreme stiffness interspersed with more compliant areas [25].

The combination of enzymatic and non-enzymatic crosslinks creates a matrix that is both highly crosslinked and mechanically heterogeneous. This heterogeneity is important for percolation behavior because regions of extreme crosslink density act as rigid nuclei that provide a template for the expansion of fibrotic tissue into adjacent areas [33].

### 6.4. Propagation: Mechanical and Biochemical Feedback

Once fibrotic tissue has crossed the rigidity percolation threshold, multiple feedback mechanisms drive progression. Mechanically, the stiff fibrotic tissue concentrates stress on adjacent normal tissue during breathing. These stress concentrations can damage alveolar epithelium, triggering the injury–repair cycle that initiates fibrosis in new regions [49]. The tissue at the leading edge of this process represents active sites of myofibroblast proliferation and matrix deposition. These foci exhibit extremely high LOX and LOXL2 expression, resulting in locally increased crosslinking [50]. As these foci deposit and crosslink the matrix, they extend the rigid percolation network into previously normal tissue.

MMPs have significantly reduced activity against heavily crosslinked collagen, and their increased expression may be counteracted by the ability of crosslinks to mask the enzymatic sites they recognize [13]. This resistance to degradation means fibrosis is essentially irreversible once established. Unlike inflammatory damage that can heal, crossing the rigidity percolation threshold creates a mechanically and biochemically stable pathological state. The tissue has reached a new equilibrium where extensive crosslinking prevents it from returning to the compliant, remodeling-competent state of normal lung tissue.

## 7. Determinants of Pathway Selection: Why Emphysema or Fibrosis?

### 7.1. Balance of Proteolysis and Crosslinking

The fundamental determinant is the relative rates of matrix degradation versus crosslinking. When protease activity exceeds the capacity for crosslink formation and repair, crosslink density decreases, moving tissue toward the degradative percolation threshold characteristic of emphysema. When crosslink formation exceeds degradation, particularly in the context of excessive matrix synthesis, tissue moves toward rigidity percolation and fibrosis.

**Figure 10:**
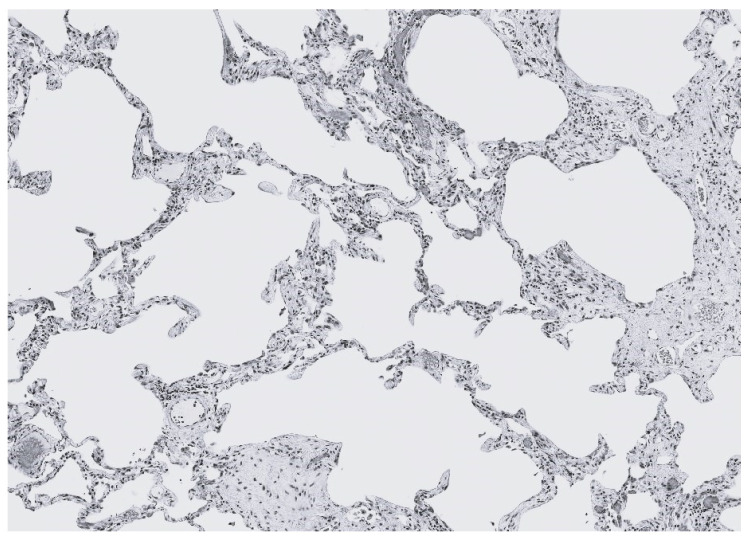
Photomicrograph of CPFE showing cystic airspaces surrounded by interstitial fibrosis.

In cigarette smoke-induced disease, the high burden of proteases and oxidative crosslink damage usually tips the balance toward degradation. However, some smokers develop combined pulmonary fibrosis and emphysema (CPFE), suggesting that in certain individuals or lung regions, both processes are not mutually exclusive and may instead be opposite ends of a spectrum determined by local protease–crosslink balance (Figure 10) [51,52]. The spatial heterogeneity in LOX levels, due to regional differences in mechanical stress, cell populations, and inflammatory milieu, may also contribute to the coexistence of both disease entities.

### 7.2. Matrix Composition and Pre-Existing Crosslink Density

The baseline state of the extracellular matrix may influence susceptibility to either type of transition. Tissue with lower crosslink density is closer to the degradative percolation threshold and may more readily develop emphysema. Genetic variations in LOX or collagen genes that affect baseline crosslink density may predispose toward one pathway or the other. The dominance of either percolation process may also be dependent on age-related changes in matrix composition and crosslinking. Older individuals have accumulated more crosslinks, which might provide some protection against degradative percolation but could also promote fibrosis by creating an initially stiff mechanical environment [53].

### 7.3. Cell Populations and Inflammatory Milieu

The specific inflammatory cell populations and cytokine profiles differ markedly between emphysema and fibrosis, and these differences exert profound, direction-specific effects on extracellular matrix (ECM) turnover. Emphysema is characterized by a predominance of neutrophils and classically activated (M1) macrophages, which release high levels of proteases such as neutrophil elastase, MMP-9, and MMP-12, along with pro-inflammatory cytokines including TNF-α, IL-1β, and IL-8 [54,55]. This inflammatory constellation drives net ECM degradation, reduces crosslink density, and pushes the alveolar network toward downward connectivity percolation, where loss of structural links leads to progressive airspace enlargement and mechanical failure.

In contrast, interstitial fibrosis is dominated by alternatively activated (M2) macrophages, TGF-β–rich cytokine environments, and the recruitment and differentiation of myofibroblasts [56]. These cells secrete collagen I and III, fibronectin, and matricellular proteins, while also producing LOX/LOXL enzymes that increase covalent crosslinking [57]. The result is a microenvironment that favors matrix accumulation, rigidity percolation, and the emergence of stiff, mechanically self-reinforcing fibrotic foci. M2 macrophages further amplify this trajectory through secretion of IL-10, CCL18, and PDGF, which sustain fibroblast activation and suppress protease activity, thereby tipping the balance toward excessive crosslink formation [58].

Understanding how the immune response is polarized in individual patients could therefore provide predictive insight into disease trajectory and therapeutic responsiveness. Biomarkers of macrophage phenotype, cytokine signatures, and protease–antiprotease balance may help identify patients at risk for rapid progression, mixed phenotypes, or atypical remodeling patterns. Moreover, interventions that modulate macrophage polarization, inhibit TGF-β signaling, or rebalance proteolytic and crosslinking forces could theoretically shift the tissue back across the percolation threshold, altering the course of disease.

## 8. Therapeutic Implications: Targeting Crosslinking and Percolation

### 8.1. Emphysema: Preventing Degradative Percolation

In emphysema, therapeutic goals include preventing crosslink loss, blocking catastrophic percolation transitions, and potentially restoring connectivity above the critical threshold. Although several different approaches have been proposed, none have been adequately tested in either preclinical or human studies.

Antioxidant strategies are designed to prevent injury by free radicals in cigarette smoke and other air pollutants. While clinical trials of simple antioxidants have been disappointing, more targeted approaches that specifically protect crosslink structures might be more effective [59]. Compounds that stabilize elastin crosslinks or prevent desmosine oxidation could maintain the elastic network above the percolation threshold. Furthermore, LOX upregulation in remaining intact tissue might enhance repair responses. Strategies to increase enzymatic crosslink formation in regions near emphysematous damage could stabilize the boundary and prevent propagation. However, this approach must be carefully controlled to prevent excessive crosslinking, which could lead to fibrosis.

MMP inhibitors have been explored but with limited success, partly because these enzymes have important homeostatic functions [60,61]. Targeted inhibition specifically at the boundaries of emphysematous regions, where mechanical stress concentrations accelerate damage, might be more effective than systemic inhibition. Localization of the process may be facilitated by attaching specific matrix binding agents to the inhibitors.

Regenerative approaches utilizing stem cells or progenitor cells aim to repair and rebuild damaged tissue [62,63]. From a percolation perspective, these approaches should not only replace cells but also restore matrix connectivity. Scaffolds that provide appropriate crosslink density and mechanical properties may be necessary to guide tissue regeneration above the percolation threshold.

### 8.2. Fibrosis: Preventing Rigidity Percolation

LOX inhibitors, particularly those targeting LOXL2, have shown promise in preclinical models and early clinical trials [64,65]. Simtuzumab, a monoclonal antibody targeting LOXL2, has been shown to reduce fibrosis in animal models, but a clinical trial in idiopathic pulmonary fibrosis was disappointing [66]. This result may reflect limitations in antibody penetration into established fibrotic tissue or treatment initiation too late in the disease course. Next-generation LOX inhibitors with better tissue penetration or administered earlier might be more effective.

Transglutaminase inhibitors represent another crosslinking intervention. Small-molecule TG2 inhibitors have demonstrated efficacy in preventing the development of fibrosis in animal models [67]. However, transglutaminases have important physiological functions, requiring novel drug delivery strategies that may involve the coupling of these inhibitors to extracellular matrix attachment molecules [68].

Another approach involves the use of compounds that cleave established crosslinks. AGE crosslink breakers have been developed for diabetes complications, and adapting these for pulmonary fibrosis could reduce matrix stiffness in established disease [69]. This strategy could be combined with anti-TGF-β therapies that reduce LOX expression and myofibroblast activation. Pirfenidone, one of the approved treatments for idiopathic pulmonary fibrosis, may work in part by downregulating TGF-β signaling and LOX expression [70].

At higher levels of scale, the use of agents that target the effects of mechanical stress could slow the progression of interstitial fibrosis. In particular, inhibition of the YAP/TAZ signaling pathway could limit cell proliferation and matrix deposition resulting from mechanotransduction feedback loops associated with fibrogenesis [71]. The activation of this pathway promotes the attachment of mechanosensitive integrins to fibroblasts, inducing myofibroblast differentiation and excessive ECM deposition. It also disrupts epithelial repair programs, impairing alveolar epithelial cell renewal, a central feature of fibrotic lung disease. Beyond fibroblasts and epithelium, YAP/TAZ signaling modulates macrophage-mediated inflammation, amplifying fibroinflammatory circuits that sustain chronic injury [72]. Collectively, these pathways position YAP/TAZ as nodal regulators linking mechanical stress, cellular plasticity, and persistent matrix remodeling in pulmonary fibrosis, making them compelling therapeutic targets under active investigation.

Understanding that emphysema and fibrosis represent opposite percolation transitions suggests that therapeutic strategies should be individualized based on assessment of which transition threatens individual patients. Biomarkers of crosslink turnover, such as urinary desmosine in emphysema or serum markers of LOX activity in fibrosis, might guide therapy selection [73,74]. Furthermore, imaging techniques that assess tissue mechanical properties could identify regions approaching critical percolation thresholds before irreversible changes occur. In particular, magnetic resonance elastography, which couples conventional MRI with low-frequency mechanical vibrations to map tissue stiffness, can produce a quantitative “elastogram” that reflects underlying biomechanical properties [75].

While the measurement and standardization of biomarker levels need further development, desmosine and isodesmosine levels in sputum and urine have been used as a real-time indicator of therapeutic efficacy in clinical trials of novel agents for the treatment of pulmonary emphysema. In a 28-day trial of aerosolized hyaluronan in COPD patients with alpha-1 antitrypsin deficiency, peptide-free desmosine and isodesmosine levels in urine were significantly reduced [76]. Furthermore, the ratio of free to peptide-bound urinary desmosine and isodesmosine in bronchoalveolar lavage fluid was markedly lower in bleomycin-induced pulmonary fibrosis compared to elastase-induced emphysema, consistent with the proliferation of these crosslinks as percolation connectivity progresses.

### 8.3. Therapeutic Limitations

The multiscale emergent properties of pulmonary emphysema and interstitial fibrosis have profound implications for developing effective therapies for these diseases [77,78]. First, they suggest that single-target molecular therapies, while conceptually elegant and successful in simpler diseases, may be fundamentally insufficient for complex chronic lung diseases. Combination approaches that simultaneously target multiple scales, including molecular pathways, cellular behaviors, tissue mechanics, and systemic factors, may be necessary to achieve meaningful clinical benefit (Table 1) [79,80,81].

Therapies might be most effective in disease when the pathological state is less entrenched and reversibility remains possible, but this requires improved early diagnosis and risk stratification. Alternatively, more aggressive interventions capable of disrupting established attractor states might be needed in advanced disease, but such approaches must be carefully designed to avoid catastrophic destabilization of remaining lung function.

**Figure 11:**
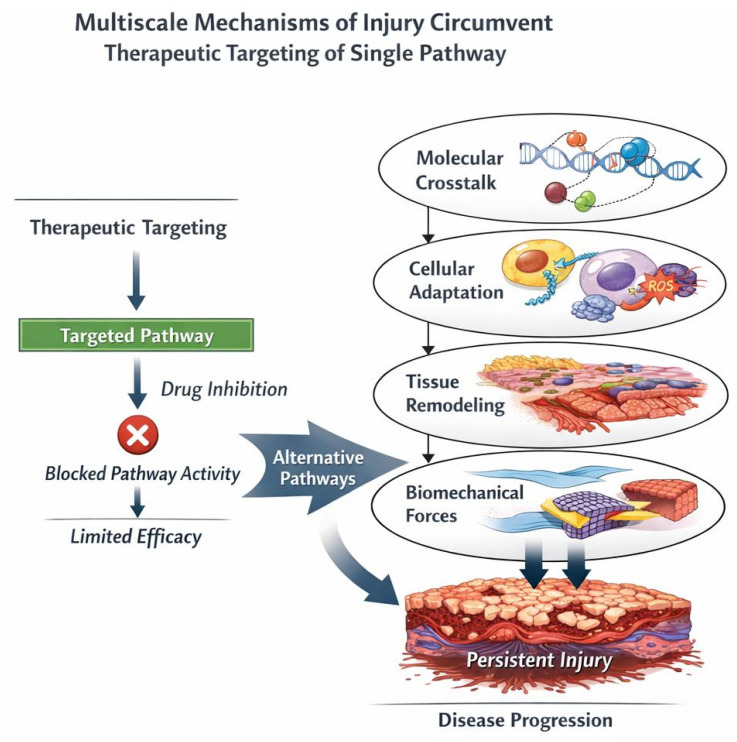
Multiscale mechanisms of injury circumvent therapeutic targeting of a single pathway. Despite molecular blockade by a targeted therapeutic agent, injury propagates through alternative signaling routes and escalates across biological scales. Arrows illustrate bypass routes and upward propagation, emphasizing that single-pathway interventions may be insufficient to halt multiscale injury progression.

These considerations highlight the importance of combination therapies that address disease at multiple scales simultaneously. For fibrosis, this might mean combining anti-crosslinking therapy with interventions targeting inflammation, epithelial dysfunction, mechanical stress, and systemic complications. For emphysema, combination of protease inhibition, anti-inflammatory therapy, and strategies to promote appropriate matrix repair might prove necessary (Figure 11).

**Figure 12:**
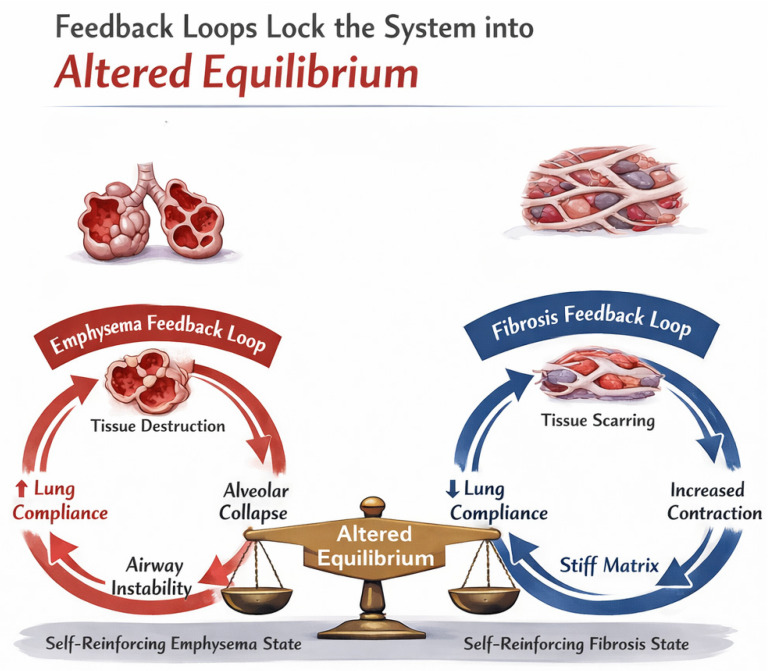
The application of game theory principles to pulmonary emphysema and interstitial fibrosis suggests that pathological feedback loops become increasingly entrenched, making these diseases more resistant to therapeutic intervention.

The application of game theory principles might also be useful in developing treatment strategies. In terms of lung disease, this theory proposes that the behavior of individual cells is not an isolated phenomenon but is instead governed by aberrant feedback loops that have become adapted to a shifting balance in percolation forces, such as mechanical stress, protease activity, and cell survival [81,82]. Under these circumstances, there is no advantage for the system to return to its original healthy state. This transformation could establish a pathological equilibrium requiring therapeutic intervention before the altered feedback loops become normalized and less amenable to reversal (Figure 12).

## 9. Conclusions

The percolation framework provides a unifying perspective on the divergent pathological outcomes of chronic lung inflammation. Emphysema and interstitial fibrosis, rather than being entirely distinct diseases, represent opposite transitions through critical percolation thresholds: one involving the loss of structural connectivity through crosslink degradation, and the other involving excessive connectivity through pathological cross-linking.

Crosslinking emerges as the central molecular mechanism governing these transitions. The balance between crosslink formation and degradation determines whether tissue maintains homeostatic connectivity, fragments into emphysematous destruction, or rigidifies into fibrotic consolidation. The types of crosslinks, enzymatic versus non-enzymatic and properly positioned versus random, further influence mechanical properties and susceptibility to percolation transitions.

Understanding these transitions as percolation phenomena explains several puzzling clinical observations, including the non-linear progression of disease, the heterogeneous spatial distribution of pathology within the lungs, and the coexistence of emphysema and fibrosis in some patients. This framework also highlights why current therapies have had limited success. Interventions that do not address the fundamental percolation dynamics cannot reverse disease once critical thresholds have been crossed. Future therapeutic strategies must target the crosslinking mechanisms and mechanical feedback loops that determine percolation transitions.

The percolation perspective ultimately reframes our understanding of chronic lung disease from a purely biochemical problem to one involving critical transitions in physical network properties. Crosslinking serves as the molecular mechanism controlling these transitions, making it both a marker of disease state and a therapeutic target. By recognizing that emphysema and fibrosis represent opposite sides of percolation thresholds, we can develop more rational, mechanism-based approaches to preventing and treating these devastating diseases. The challenge ahead is to translate these insights into clinical tools that can detect approaching transitions and interventions that can stabilize lung tissue within the homeostatic range between degradative and rigidity percolation thresholds.

## Figures and Tables

**Table 1:** Multiscale Changes in Pulmonary Fibrosis and Interstitial Fibrosis.

Scale/Feature	Pulmonary Emphysema	Interstitial Fibrosis
**Molecular Scale**		
Primary Process	Proteolytic degradation of ECM	Excessive ECM synthesis and deposition
Key Molecules	Matrix metalloproteinases (MMPs), elastases, oxidants	TGF-β, collagen I/III, fibronectin, α-SMA
Elastin Status	Degraded and fragmented	Preserved or increased
Collagen Content	Decreased or normal	Markedly increased (2–5x normal)
**Cellular Scale**		
Cell Types Involved	Neutrophils, macrophages, inflammatory cells	Myofibroblasts, fibroblasts, epithelial cells
Cellular Response	Inflammation-driven destruction	Aberrant wound healing response
Alveolar Epithelium	Loss of capillary-alveolar interface	Type II pneumocyte hyperplasia, epithelial injury
**Tissue Scale (Microscopic)**		
Alveolar Structure	Enlarged airspaces, wall destruction	Collapsed/obliterated airspaces, thickened walls
Interstitium	Thinned or absent	Markedly thickened with scar tissue
Architecture Pattern	Loss of normal architecture	Honeycombing, architectural distortion
Crosslink Percolation	Below threshold: isolated clusters	Above threshold: spanning network
**Mechanical Properties**		
Tissue Compliance	Increased (hyperelastic, floppy)	Decreased (stiff, rigid)
Elastic Recoil	Reduced (loss of elastic fibers)	Reduced (increased stiffness)
Stress Distribution	Heterogeneous, stress concentrations	Transmitted through percolated network
**Organ Scale (Macroscopic)**		
Lung Volume	Increased (hyperinflation)	Decreased (restrictive pattern)
CT Appearance	Low attenuation areas, bullae	Reticular opacities, ground-glass, honeycombing
Distribution	Upper lobe predominant (typically)	Lower lobe and peripheral predominant
Surface Appearance	Overinflated, pale, bullous	Shrunken, firm, nodular
**Functional/Clinical Scale**		
Primary Defect	Obstructive (air trapping)	Restrictive (reduced expansion)
FEV1/FVC Ratio	Decreased (<0.70)	Normal or increased
DLCO	Decreased (loss of surface area)	Decreased (thickened membrane)
Gas Exchange	Impaired (V/Q mismatch)	Impaired (diffusion limitation)
Breathlessness Pattern	Progressive with air trapping	Rapid, shallow breathing
Reversibility	Largely irreversible structural damage	Progressive and irreversible scar

## Data Availability

No new data were created or analyzed in this study.
